# The Use and Abuse of the X-Ray in Surgery

**Published:** 1899-04

**Authors:** J. McF. Gaston

**Affiliations:** Atlanta, Ga.


### THE USE AND ABUSE OF THE X–RAY IN SURGERY.*

By J. McF. GASTON, Jr., A.M., M.D.,

Atlanta, Ga.

The use of the X-ray has been very wide and varied within the last three years. In the recent war with Spain our military surgeons had occasion to use it in every important gunshot wound. Viewed in the recent improved condition, the discovery of Prof. Roentgen ranks with the thirteen great discoveries and inventions of the nineteenth century, and is classed with antiseptic surgery by the writer who answers the column headed "Mail-Bag" in the *Christian Herald.* The recent advances of surgery owe much to the teachings of Lister; but none will disagree in the practical application of the discovery of Roentgen to surgery. Among the great and lasting strides of surgery are the location of foreign

---

*Read before the Atlanta Society of Medicine, March 2, 1899.

bodies, the great and important results in the observation of fractures, and the recent results in detecting cavities in the lungs. In the location of tumors, the X-ray is invaluable.

Among the necessary apparatus in the Johns Hopkins Hospital, and the Jefferson Medical Hospital, will be found the X-ray inparatus.

Many of our members in the Atlanta Society of Medicine must have been impressed with a recent catalogue from Germany giving all the electrical apparatus, and among them X-ray machines or coils having the capacity to produce a spark of twenty-eight inches.

Great improvements in the technique of X-ray apparatus have taken place, so that the heart can be seen to beat, and the lungs to move with each respiration.

For the ordinary routine work of the surgeon in the examination of ankylosed joints, in obscure cases of gall-stones, urethral or vesical calculi, or renal calculi, a diagnosis may be reached by the use of the fluoroscope and the skiagraph. The quickest, and often the readiest, means of ascertaining whether the fragments of a fracture which have been placed in apposition have remained intact, consists in the view with a fluoroscope of the seat of the fracture; and this may be equally satisfactory whether the dressing is plaster of Paris, or pasteboard or wood.

In the majority of cases no change need be made, and the satisfaction afforded by the examination is ample recompense for the outlay or trouble.

The serious problems of the present day are being rapidly solved, and the most marvelous dexterity has been shown. Among the earlier feats was the extraction of a jackstone from the esophagus.

In the further experience of Dr. J. William White, the X-ray has been very serviceable. I might also mention Drs. Maurice H. Richardson, W. W. Keen, and Abbe.

The static machine is used by some surgeons, while the Rumkorff coil and storage battery is used by others. Abbe has had very satisfactory results, and here I will read the main parts of a letter from Dr. M. H. Richardson of Boston.

Of great importance is the portability of the apparatus. In this connection the storage battery is superior to all others. It was found that the trouble with transports in the Spanish-American war was no barrier to the packing and safe removal of the large X-ray apparatus used by Prof. Gray for Dr. Senn and other surgeons.

In the rapid process for photography, the chemicals for development, fixing, etc., were carried in tubes in the solid state. The sensitive films were ready packed in light-tight envelopes, the only necessary material which could not be carried for any distance being a supply of water.

In private office practice, a storage battery of four cells with ten amperes and ten volts and of 100 ampere-hours, has proved satisfactory to me. The battery can be charged with a continuous current, and there should be a resistance box to diminish the current of 110 volts to six or eight volts; and the use of two 52-volt lamps has been suggested. In the more extensive apparatus in hospitals, a larger storage battery in a special arrangement for using the street current has been found available.

[N. B.—In our office five 32-candle power lamps of 110 volts give $4\frac{3}{4}$ amperes in charging the storage batteries. The lamps are arranged in multiple on the negative wire.]

No danger from the apparatus as generally prepared need be apprehended. In the majority of cases, all sparking through the Crookes tube may be avoided by a condenser.

The abuse of the X-ray consists in the distortion of images for medico-legal purposes; in the substitution of the results of its information for the more valuable information to be gained by the other means at the surgeon's disposal.

In the therapeutical effects, no consensus of opinion has been reached, but some experiments are made to prove its bactericidal properties.

In the most careful cases of the location of foreign bodies, such as bullets, the telephone probe must be resorted to for final discovery of the ball.

The serious results of the burn from the X-ray are due to an abuse of the ray, either in too close proximity to the Crookes tube, longer exposure than necessary, or filthy skin. In no case have I found any burn.

2

Among the abuses of the X-ray may be mentioned its relegation to the sphere of laymen and chemists in place of the thorough knowledge of its direct manipulation by the surgeon himself.

However, it must be said that it is more valuable as skilfully and scientifically employed than by novices or amateurs.

Nothing but a knowledge of the ordinary principles of electricity need be known to master its details. A low-reading portable volt-meter for the purpose of testing the storage-batteries will enable the surgeon to determine the voltage of each separate cell in the battery, and to determine the necessity for charging the same, while the actual life of the cell is lengthened by a cautious and systematic regard for the state of each element and the constant use of the electricity therein generated.

Of incalculable benefit to one desiring to understand the subject of storage batteries is a visit to the telegraph or telephone office, where a switch-board changing some for certain other cells, and where various connections are made for charging these batteries, by simply turning a handle at specified times.

In the matter of selecting a Crookes tube, almost any good tube may be used; but there should be a self-adjusting vacuum, so that the moisture necessary in the tube may be kept up by potassium oxide (caustic potash).

In no case should a plate used for photography be expected to have a distinct shadow unless the object be a thickness of at least one-third the distance between the plate and the Crookes tube. For the hand a distance of about three inches from the plate and an exposure of a minute is sufficient, while for a leg, a foot or more; a thigh, eighteen inches; and the body, twenty-one or more inches. For body pictures a static machine is not advisable.

We have had some cases in surgery calling for the X-ray; and among them a child who had swallowed a metallic whistle such as the one exhibited. The location of the foreign body was made both with the fluoroscope and with this picture I pass around.

The presence of Dr. Hunter McGuire, of Richmond, enabled us to avail ourselves of his experience in attempting to extract it through the mouth; but all efforts in this direction proving futile, the whistle was dislodged from about the region of the sterno-clavicular articulation—of course being in the esophagus but at this level—pushed into the stomach and was passed per *vias naturales* in about eighteen days.

In the same way a bullet has been located in a boy's back, on a level between the sixth and seventh ribs, but the depth is not so easy to ascertain. The bullet is still located about where it was three years ago.

Another case was a fracture, which I have mentioned at a previous meeting; also a bullet in the hip has been located by a skiagraph.

